# Recent Progress of Low Dielectric and High-Performance Polybenzoxazine-Based Composites

**DOI:** 10.3390/polym15193933

**Published:** 2023-09-29

**Authors:** Zexu Fan, Bo Li, Dengxun Ren, Mingzhen Xu

**Affiliations:** School of Materials and Energy, University of Electronic Science and Technology of China, Chengdu 610054, China; fanzexu150@163.com (Z.F.); uestc_lb87@163.com (B.L.); rendenxun2008@126.com (D.R.)

**Keywords:** low dielectric, polybenzoxazine, copolymerization, nanoparticles

## Abstract

With the rapid advancement of intelligent electronics, big data platforms, and other cutting-edge technologies, traditional low dielectric polymer matrix composites are no longer sufficient to satisfy the application requirements of high-end electronic information materials, particularly in the realm of high integration and high-frequency, high-speed electronic communication device manufacturing. Consequently, resin-based composites with exceptional low dielectric properties have garnered unprecedented attention. In recent years, benzoxazine-based composites have piqued the interest of scholars in the fields of high-temperature-resistant, low dielectric electronic materials due to their remarkable attributes such as high strength, high modulus, high heat resistance, low curing shrinkage, low thermal expansion coefficient, and excellent flame retardancy. This article focuses on the design and development of modification of polybenzoxazine based on low dielectric polybenzoxazine modification methods. Studies on manufacturing polybenzoxazine co-polymers and benzoxazine-based nanocomposites have also been reviewed.

## 1. Introduction

The electronic information industry has witnessed rapid growth, particularly with the advent of the 5G era. As a result, there has been an unprecedented surge in research focus on high-performance and low dielectric materials, essential for meeting the demanding requirements of the high-end electronic information sector [1,2,3]. The advancement of ultra-large-scale integrated circuits has necessitated higher performance standards for low dielectric materials. These requirements include exceptional strength, elevated thermal stability, minimal hygroscopicity, low thermal swelling coefficient, and strong adhesion properties [4]. In general, low dielectric materials have dielectric constants below 3.9, which fall between the values for air and silica. These materials are commonly employed in microelectronics. Low dielectric polymer materials, such as polytetrafluoroethylene (PTFE) [5], liquid crystal polymers (LCP) [6], and polyimide (PI) [7,8], are extensively utilized in electrical and electronic engineering, printed circuit boards and communication materials due to their notable processability, thermal stability, and electrical insulation properties [9]. Polytetrafluoroethylene exhibits a comparatively low dielectric constant due to its highly symmetric molecular structure and the presence of low polar C−F bonds. However, its practical applications are restricted due to its inferior mechanical strength and high expansion coefficient [10]. Polyimide, on the other hand, demonstrates remarkable mechanical properties, good processability, high-temperature resistance, and low dielectric characteristics. Nevertheless, its limited water and alkali resistance hampers its use in low dielectric applications [11]. Additionally, phenylpropylene cyclobutene resins, which also possess low dielectric constants, present challenges in controlling the film formation process, often yielding brittle cured films [9,12]. Polybenzoxazines exhibit remarkable properties such as low cure shrinkage [13], high modulus [14,15], low coefficient of thermal expansion [16], exceptional heat resistance [17,18], and adequate flame retardancy [19,20]. These desirable attributes are attributed to the Mannich bridge structure and hydrogen bonding present in the molecular composition of polybenzoxazines [21]. These advantages make benzoxazine resins promising for use in high-end electronic information materials. However, benzoxazine resins need to be cured at moderately high temperatures, and the inherent polymerization mechanism of benzoxazine resins causes them to be brittle [22]. The good news is that due to the flexible molecular design of benzoxazines, the brittleness problem can be solved by introducing flexible chains into benzoxazines, and reactive chemical groups can be introduced to reduce the polymerization temperature [23,24,25,26]. Based on this, benzoxazine resins have attracted the research interest of a wide range of scholars in the field of high-temperature resistant high-performance low dielectric materials.

The dielectric constant decreases as the molecular polarizability and the number of polarized molecules per unit volume decrease [27]. Consequently, the dielectric constant can be reduced by increasing the free volume of polymer material and decreasing the molecular polarization rate. The methods for increasing the free volume in polymeric materials encompass various strategies such as incorporating fluorine atoms using molecular design [28,29] and hyperbranched molecular chains [30], preparing polymers as mesoporous materials [31,32], and introducing mesoporous nanoparticles [33]. Decreasing the molecular polarizability can be achieved via molecular design approaches like incorporating C−F bonds with smaller dipole moments [29], siloxane chains [34], and alkyl chains [35], as well as via blending and copolymerization with low dielectric polymeric materials [36,37]. Furthermore, it is known that the lower hygroscopic nature of dielectric materials can increase their dielectric constant by enhancing orientation polarization within the matrix due to moisture presence [38,39]. Consequently, reducing the water absorption of a material is feasible by introducing less polar functional groups, simultaneously reducing its dielectric properties. The molecular design of low dielectric benzoxazines, the design and preparation of low dielectric benzoxazine-based copolymer resins, and low dielectric benzoxazine-based nanocomposites are reviewed based on previous studies by scholars and general low dielectric modification methods.

## 2. Low Dielectric Fluorinated Polybenzoxazines

**Figure 1:**
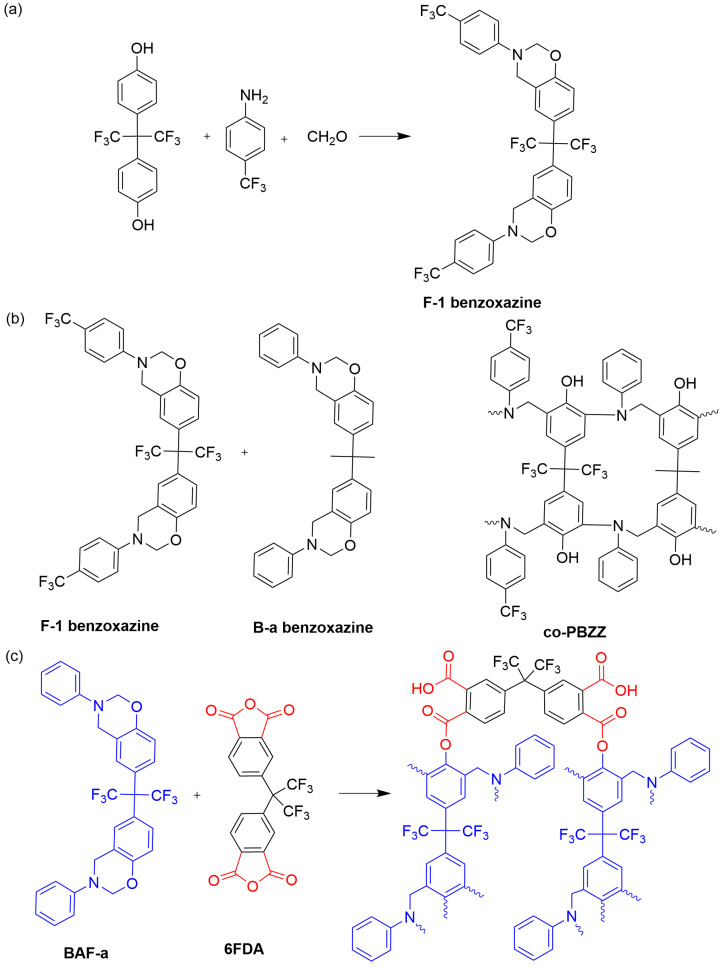
(**a**) The synthesis of F-1 benzoxazine [40]. (**b**) The ring opening process of co-PBZZ [40]. (**c**) A possible copolymerization between BAF-a and 6FDA [41].

The strong electronegativity of the fluorine atom in C−F bonds results in low polarizabilities compared to C−C bonds. Additionally, trifluoromethyl groups increase the free volume within the molecule, further decreasing its dielectric properties [28]. These factors contribute to the widespread popularity of fluorinated benzoxazines. Based on their distinct chemical structures, benzoxazine monomers can be divided into phenolic and amine benzoxazines. Phenolic benzoxazine monomers are typically synthesized by combining bisphenols, amines, and formaldehyde. To obtain fluorinated polybenzoxazines, fluorination can be applied to the bisphenol, the amine, or both components of the structure. As early as two decades ago, Su et al. [40] pioneered incorporating trifluoromethyl groups into bisphenol A and aniline, synthesizing fluorinated benzoxazine monomers designated as F-1 benzoxazine (Figure 1a). These monomers were subsequently copolymerized with fluorine-free benzoxazine monomer, denoted as B-a benzoxazine, allowing for the preparation of low dielectric benzoxazine resin via the precise modulation of the monomer ratio, termed as co-PZZ (Figure 1b). Remarkably, the co-PZZ resin with an F-1:B-a weight ratio of 1:1 exhibited a dielectric constant of 2.36 and a minimal dielectric loss of 0.0044 at a frequency of 0.1 MHz. Meanwhile, robust C−F bonds within the polymer structure confer thermal stability enhancement. Building upon these advancements, Pattharasiriwong et al. [41] extended the utilization of fluorinated bisphenol-based benzoxazine (BAF-a) by copolymerizing it with fluorinated dicarboxylic anhydride (6FDA) in various ratios (Figure 1c). Their study demonstrated that the incorporation of BAF-a facilitated the formation of ester bonds, which not only enhanced the toughness of the polymer but also increased the cross-linking density. Notably, the copolymer of BAF-a:6FDA with a 1:1 mole ratio exhibited a dielectric constant of 2.61 at a frequency of 0.1 MHz. These findings underscore the potential of such fluorinated polybenzoxazines in developing low dielectric materials with improved properties in the electronic information industry.

**Figure 2:**
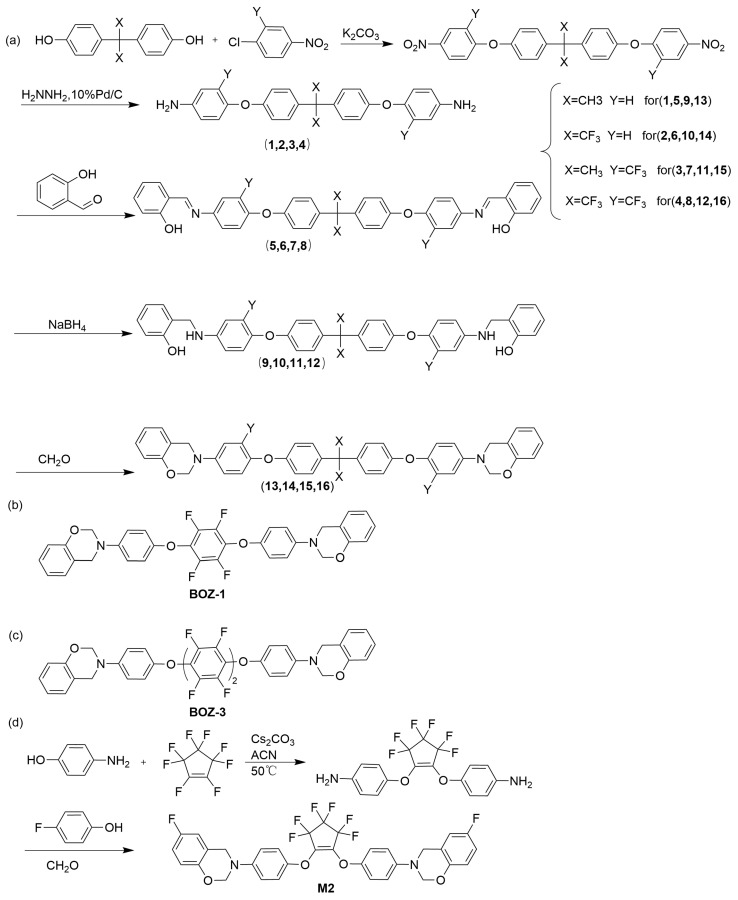
(**a**) The synthesis of 13,14,15,16 [43]. (**b**) The chemical structure of BOZ-1 [44]. (**c**) The chemical structure of BOZ-3 [45]. (**d**) The synthesis of M2 [46].

Aromatic amine-type benzoxazine monomers can be synthesized by condensing aromatic diamines, phenols, and formaldehyde. However, this conventional method often yields low quantities of the desired monomers. An alternative synthetic approach involves the reductive amination of bisphenols containing azomethine groups, followed by condensation with formaldehyde to produce aromatic amine-type benzoxazine monomers. Despite the additional steps involved in the imine reduction–condensation process, it offers a straightforward and efficient means to obtain these monomers in high yields [42]. Lin et al. [43] synthesized three fluorinated benzoxazines (Figure 2a) using the method above from three fluorinated aromatic diamines and conducted a comparative analysis of three polybenzoxazines with different fluorinated structures. The presence of bulky −CF_3_ substituents resulted in an increased free volume, which was expected to lead to a lower dielectric constant of P(16) compared to P(14). Surprisingly, the dielectric constant of P(16) was slightly higher than that of P(14). It was discovered that the neighboring −CF_3_ substituent spatially obstructed the ring opening of the benzoxazine, leaving some unreacted oxazine rings. This unreacted portion increased the polybenzoxazines’ polarity, resulting in a higher dielectric constant for P(16). Kobzar et al. [44] synthesized an aromatic amine-type benzoxazine (BOZ-1) using 1,4-tetrafluorobenzene (TFB) (Figure 2b). The resulting polybenzoxazine exhibited high crosslink density, remarkably low dielectric properties, and frequency stability. Specifically, at frequencies ranging from 10^2^ Hz to 10^5^ Hz, the dielectric constant remained stable at around 2.3, while the dielectric loss was maintained at approximately 0.004. Expanding upon these findings, Kobzar et al. [45] further synthesized an aromatic amine-type benzoxazine (BOZ-3) with a central unit of 4,4-octafluorobiphenylene dioxyphenylene (Figure 2c). This polybenzoxazine also displayed favorable low dielectric properties. Additionally, the Introduction of highly electronegative fluorine atoms decreased surface free energy and increased hydrophobicity, further reducing the polymer’s effective dielectric constant due to the presence of water, which will enhance the orientation polarization of these polybenzoxazines throughout the matrix [38,39]. Wu et al. [46] synthesized fluorinated aromatic diamines via the addition-elimination reaction mechanism of perfluoro cyclopentene (OFCP) with 4-aminophenol. They further prepared aromatic amine-type fluorinated benzoxazines (M2) via a one-step synthesis involving condensation with 4-fluorophenol and paraformaldehyde (Figure 2d). The resulting arylamine-type fluorinated benzoxazine resin exhibited favorable low dielectric properties, with a dielectric constant of 2.61 at a frequency of 1 MHz, while demonstrating good frequency stability.

## 3. Bio-Based Low Dielectric Polybenzoxazine

**Figure 3:**
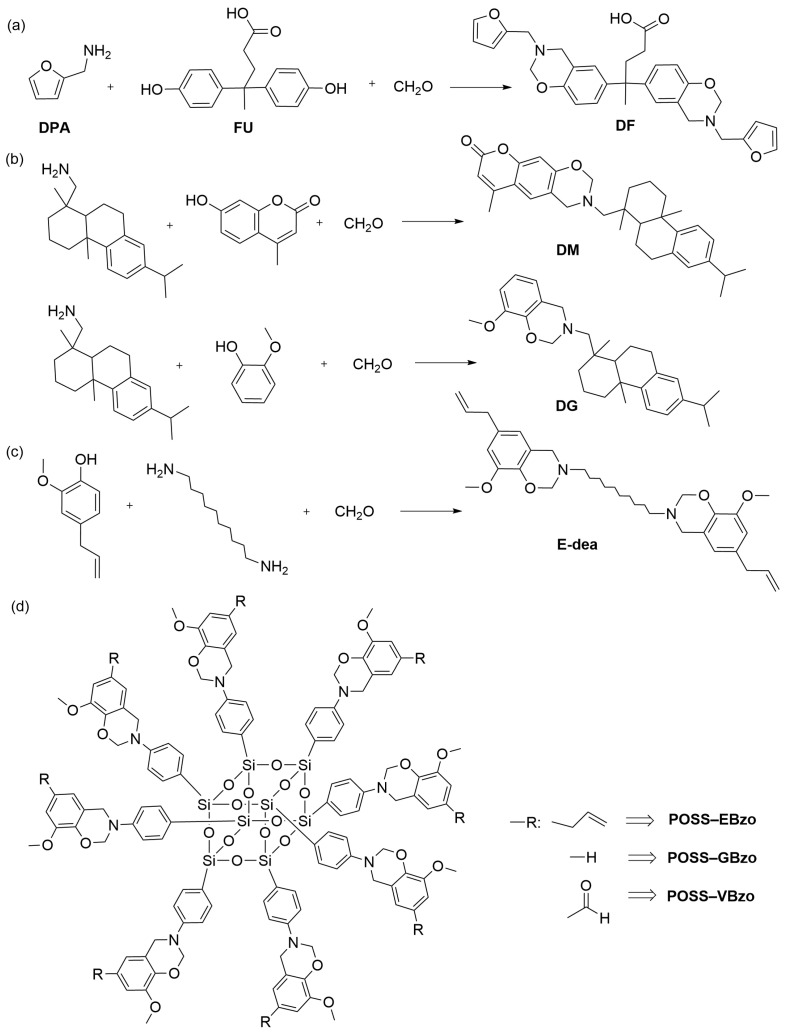
(**a**) The synthesis of DF [47]. (**b**) The synthesis of DM [48]. (**c**) The synthesis of DG [35]. (**d**) The chemical structure of POSS–EBzo/POSS–GBzo/POSS–VBzo [49].

Petroleum resources currently serve as the foundation of the chemical industry. However, with the escalating environmental challenges and potential future scarcity of petroleum energy, researchers are growing interested in developing materials derived from renewable biobased sources. Feng et al. [47] synthesized biobased phenolic benzoxazines (DF) using a one-pot method and diphenolic acid (DPA), furfural amine (FU), and paraformaldehyde as raw materials (Figure 3a). The carboxyl group in DPA and the furan ring in FU underwent esterification and electrophilic substitution reactions, respectively. These reactions resulted in a decrease in polar groups and an increase in cross-linking degree. The enhanced cross-linking density not only improved thermal stability but also restricted the movement of polar groups along the molecular chain, leading to a decrease in the dielectric constant. Additionally, the furan ring reduced molecular polarity by forming intramolecular hydrogen bonds, further contributing to the reduction in the dielectric constant. Ultimately, the polybenzoxazine resin achieved a remarkably low dielectric constant of 2.90 at 15 GHz. Liu et al. [48] synthesized fully biobased benzoxazines, including benzoxazine DM and benzoxazine DG, using dehydroabietic amine, 4-methylumbelliferyl ketone, guaiacol, and paraformaldehyde (Figure 3b). They prepared polybenzoxazines PDM and PDG using these benzoxazines, respectively. A large phenanthrene ring structure in these polybenzoxazines increased the free volume. Additionally, the phenanthrene ring acted as an electron-donating group, reducing the polarity of C−N bonds and subsequently lowering the dielectric constants of the polybenzoxazines. At 25 °C and 1 kHz, the dielectric constants of PDM and PDG were 3.15 and 3.30, respectively. Remarkably, both biobased polybenzoxazines exhibited excellent low dielectric properties at a high temperature of 200 °C. Notably, due to 4-methylumbelliferone larger structure, 4-methylumbelliferone further increased the free volume, resulting in PDM displaying a lower dielectric constant compared to PDG. Sha et al. [35] developed a bio-based benzoxazine named E-dea (Figure 3c) using eugenol and 1,10-diaminodecane, along with paraformaldehyde, and by using a solvent-free method. To mitigate the potential negative impact of long decane chains on the heat resistance of the polymer, the researchers copolymerized E-dea with bismaleimide (BMI). The copolymerization process involved the initial polymerization of the allyl group of eugenol with maleimide, followed by the occurrence of the ring-opening polymerization of benzoxazines and Diels–Alder reaction when the temperature was raised above 200 °C. The copolymers exhibited reduced dielectric properties due to the low polarity of the flexible long decane chains. The resulting poly(E-dea/BMI) exhibited a dielectric constant of 2.79 at 1 MHz. Periyasamy et al. [49] synthesized three bio-based benzoxazines, which named POSS-EBzo, POSS-Gbzo, and POSS-Vbzo (Figure 3d). They then utilized these benzoxazines to synthesize three fully renewable polybenzoxazine nanocomposites (POSS-EPBZ, POSS-GPBZ, and POSS-VPBZ) with exceptional properties. The dielectric constants of POSS-EPBZ, POSS-GPBZ, and POSS-VPBZ were remarkably low, measuring only 1.98, 1.85, and 1.88, respectively, at 1 MHz. This can be attributed to the low polarity of the POSS molecule itself, combined with the homogeneous distribution of POSS within the matrix, which increased the free volume of the polybenzoxazines. These findings demonstrate the potential of these bio-based polybenzoxazine as promising materials with low dielectric constants.

## 4. Other Low Dielectric Benzoxazines

**Figure 4:**
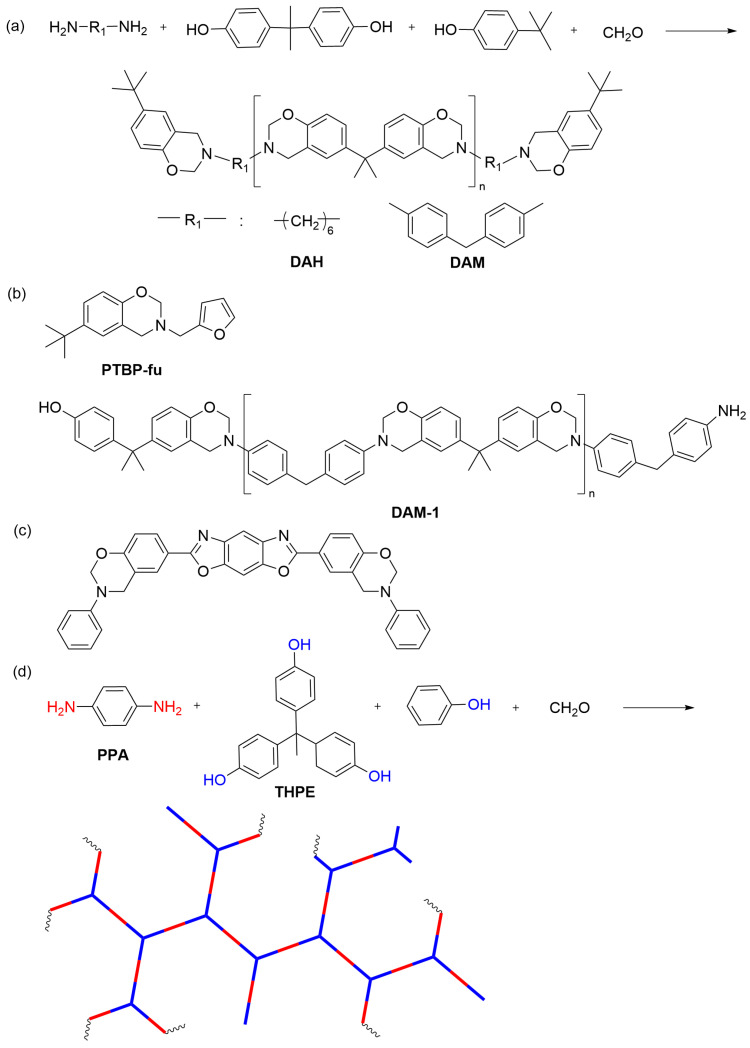
(**a**) The synthesis of benzoxazine ring-containing prepolymers with large hydrocarbon end groups [50]. (**b**) The chemical structure of PTBP-fu and DAM-1 [51]. (**c**) The chemical structure of Benzoxazines containing benzoxazole groups [52]. (**d**) Schematic structure of hyperbranched benzoxazine prepolymers [53].

Besides the mentioned molecular design strategies for achieving low dielectric benzoxazines, other approaches include incorporating large hydrocarbon groups, bulky groups, and heterocycles and designing hyperbranched benzoxazines. Chen et al. [50] synthesized two prepolymers for polybenzoxazines, one based on long-chain aliphatic diamine (DAH) and the other based on rigid aromatic diamine (DAM) (Figure 4a). The aliphatic diamine-based polybenzoxazines showed lower dielectric constant and dielectric loss than the aromatic diamine-based benzoxazines due to DAH’s low polarity alkane chain. The prepolymerization followed by curing increased the crosslink density of polybenzoxazine, thereby restricting the movement of polar groups and reducing the dielectric constant. Additionally, the bulky −C(CH_3_)_3_ group effectively increased the free volume and decreased the dipole density, resulting in a lower dielectric constant. Consequently, both benzoxazines achieved low dielectric constants (2.26/2.66, 10 GHz) and low dielectric losses (0.0047/0.0091, 10 GHz) at high frequencies. Zeng et al. [51] synthesized a benzoxazine (PTBP-fu) with bulky groups and an active furan ring. They then copolymerized it with a benzoxazine prepolymer (DAM-1) (Figure 4b) to create a polybenzoxazine with a multi-structured network. This network had denser regions near the furan ring and looser regions near the bulky groups. The loose network portions increased the free volume, while the dense network portions increased the cross-linking density, effectively restricting the movement of molecular chains and limiting the polarization of polar groups. The bulky group (−C(CH_3_)_3_) significantly increased the free volume, and the Introduction of the furan ring reduced molecular polarity while increasing the cross-linking density. These factors synergistically contributed to a reduction in the dielectric constant of the polybenzoxazine. Additionally, the incorporation of low-polarity and bulky hydrocarbon groups weakened the polarization of space charge and electrons, leading to a decrease in dielectric loss. Experimental data revealed that the PTBP-fu/DAM-1 (5:1) copolymer demonstrated low dielectric constant (2.81) and dielectric loss (0.0067) at a high frequency of 10 GHz. Zhang et al. [52] developed a benzoxazine with a benzoxazole moiety using a non-solvent method (Figure 4c). The resulting polybenzoxazine displayed a low dielectric constant of 2.4 at a high temperature of 200 °C and a high frequency of 10 MHz. Cai et al. [53] synthesized two hyperbranched benzoxazine prepolymers (Figure 4d) by carefully controlling the addition time of the capping agent. The raw materials included 1,1,1-tris(4-hydroxyphenyl)ethane (THPE), p-phenylenediamine (PPA), paraformaldehyde, and phenol as the capping agent. Subsequently, polybenzoxazines were prepared. The study demonstrated that both polybenzoxazines exhibited low dielectric constants ranging from 2.15 to 2.21, as well as low dielectric losses ranging from 0.0010 to 0.0208 in the high-frequency range of 2–18 GHz.

## 5. Design of Low Dielectric Benzoxazine-Based Copolymers

**Figure 5:**
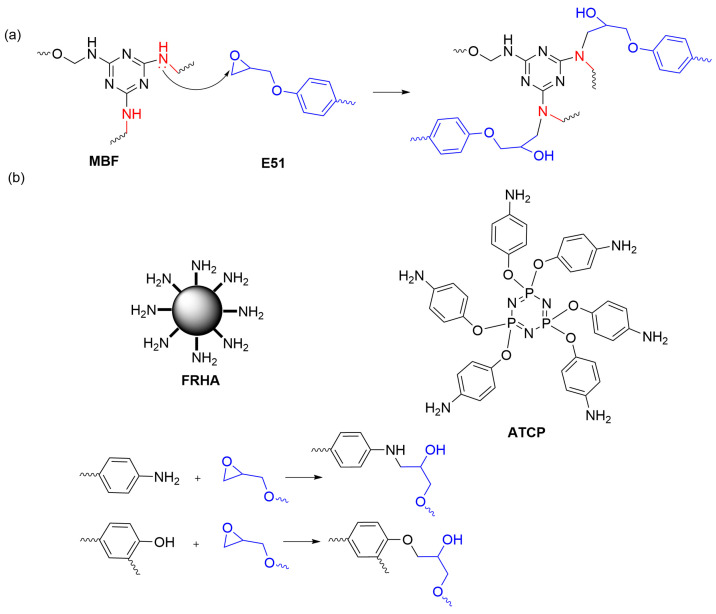
(**a**) Curing reaction between MBF and E51 [54]. (**b**) Schematic structure of FRHA; The chemical structure of ATCP; curing reactions between reactive amino and hydroxyl groups and epoxy groups [55].

Blending and copolymerizing polybenzoxazines with other polymers offer cost-effective and simplified approaches to designing low dielectric benzoxazine resins. These strategies not only reduce costs but also bring unexpected synergistic effects, leading to improved dielectric and other properties of polybenzoxazines. Ye et al. [54] synthesized benzoxazine (MBF) using melamine and furanamine as amine sources. Copolymerization with epoxy resin (E51) was performed to produce copolymers (Figure 5a). The study demonstrated that by adjusting the MBF: E51 weight ratio to 1:0.6, the copolymer achieved a reduced dielectric constant of 2.94 while simultaneously enhancing its toughness. Incorporating epoxy resin in the copolymer resulted in a depletion of polar amino groups in benzoxazine, leading to decreased molecular polarity. This, in turn, increased the crosslink density of the polymer and contributed to the reduction in the dielectric constant. Krishnadevi et al. [55] prepared a copolymer (ATCP/FRHA/Bz-Ep) by incorporating telamidophosphonitrile (ATCP) and aminosiloxane-functionalized rice husk ash (FRHA) into a copolymer of benzoxazine and epoxy resin (Bz-Ep) (Figure 5b). This approach aimed to enhance the copolymer’s flame retardancy and dielectric properties. The copolymer with 15 wt% ATCP and 5 wt% FRHA showed an exceptionally low dielectric constant of 1.62 within the frequency range of 1 MHz to 20 MHz. The observed reduction in dielectric constant was attributed to the multiple cross-linking densities and intramolecular hydrogen bonding in the copolymer, which reduced the overall polarity of the polymer network. Additionally, the presence of low-polarity siloxane structures further contributed to reducing the copolymer’s dielectric constant.

**Figure 6:**
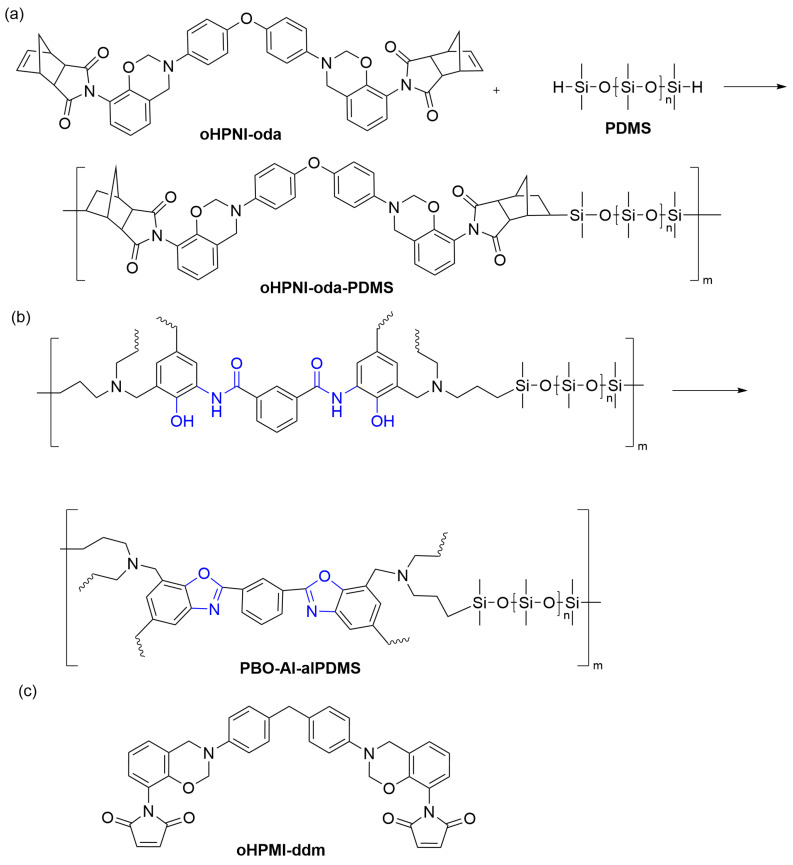
(**a**) The synthesis of oHPNI-oda-PDMS [56]. (**b**) Generative reactions of benzoxazole structures in PBO-AI-alPDMS [57]. (**c**) The chemical structure of oHPMI-ddm [58].

Zhang et al. [56] developed a benzoxazine (oHPNI-oda) containing imide and o-norbornene, which was copolymerized with polydimethylsiloxane (PDMS). The double bond in o-norbornene reacted with PDMS to form a prepolymer (oHPNI-oda-PDMS) (Figure 6a). This prepolymer was subsequently crosslinked via a ring-opening reaction of the benzoxazine ring, forming a copolymer, poly(oHPNI-oda-PDMS). The copolymer, oHPNI-oda-PDMS, exhibited very low dielectric constants (2.36–2.29) and low dielectric losses (0.007–0.002) over a wide frequency range (1 Hz–1 MHz). Incorporating low-polarity PDMS reduced the overall molecular polarity, while the crosslinked network formed by the combination of rigid aromatic and flexible PDMS segments reduced the dielectric constant by increasing free volume and decreasing water absorption in the copolymers. Yang et al. [57] synthesized a copolymer (PBO-AI-alPDMS) by copolymerizing polydimethylsiloxane (PDMS) with benzoxazine (AI-al), which has allyl and amide-bond, followed by high-temperature curing that converted the o-hydroxyl and amide groups into benzoxazole structures (Figure 6b). The obtained data revealed that the copolymer exhibited a remarkably low and stable dielectric constant of 2.51–2.13 within the frequency range of 1 Hz–1 MHz. The low dielectric constant was attributed to the depletion of polar hydroxyl groups via the formation of benzoxazole, as well as the incorporation of PDMS, which reduced the molecular polarity and water absorption of the polymer. Zhang et al. [58] synthesized a benzoxazine (oHPMI-ddm) (Figure 6c) that contained a maleimide group at the end. A benzoxazole structure was formed by utilizing an amide bond in the adjacent bismaleimide (BMI). Incorporating the bismaleimide group increased the crosslinking density, and the formation of benzoxazole between the maleimide and hydroxyl groups converted the polarizable phenolic hydroxyl group into a less polarized benzoxazole functionality. This conversion led to a significantly low dielectric constant in this polybenzoxazine.

**Figure 7:**
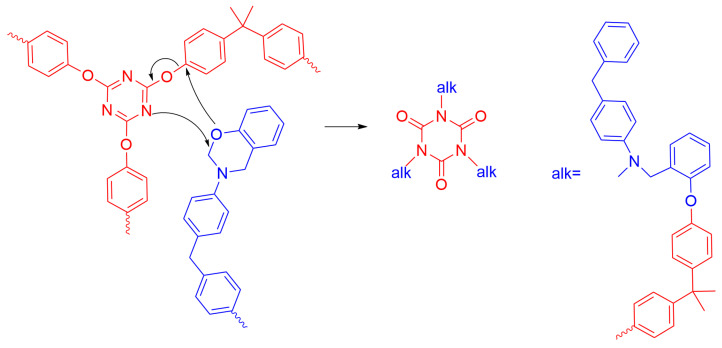
Mechanism of copolymerization reaction between BACY and Pddm [59].

Lin et al. [59] achieved cross-linking curing by blending and copolymerizing bisphenol A bis(cyanurate) (BACY) with diaminobenzoxazine (Pddm). The copolymerization occurred via the co-reaction of the triazine ring with the benzoxazine ring, forming alkyl isocyanurate and diphenyl ether bonds (Figure 7). The less polar diphenyl ether moiety contributed to a lower dielectric constant, and the copolymer exhibited the lowest Dk values of 2.78/1 GHz when the mass ratio of BACY to Pddm was 1:0.3.

The dielectric constant can be achieved by increasing crosslink density, depleting polar hydroxyl groups, forming intramolecular hydrogen bonds, introducing low-polarity groups, and incorporating large-site-resistance groups in copolymerizing. These approaches indirectly reduce the polarity of polybenzoxazines or decrease the density of dipole moments. Additionally, increasing material voids can directly reduce the density of dipole moments in polybenzoxazine resins. Su et al. [31] synthesized multihollow polybenzoxazines by copolymerizing poly(3-caprolactone) (pa-PCL) of different molecular weights with benzoxazines using poly(benzoxazine) (PBZZ) of type B-a as a substrate. The multihollow polybenzoxazines were obtained by washing away the pa-PCL in a weak alkaline solution. It was observed that adding 25 wt% pa-PCL resulted in a dielectric constant of 1.95 at 0.1 MHz for the multihollow polybenzoxazines.

## 6. Low Dielectric Benzoxazine-Based Nanocomposites

**Figure 8:**
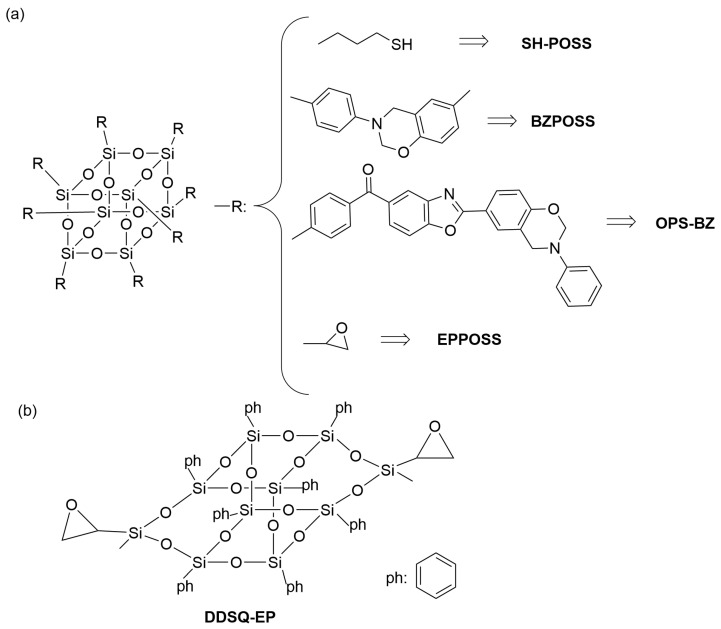
(**a**) The chemical structures of various POSS [62,63]. (**b**) The chemical structures of DDSQ-EP [64].

**Figure 9:**
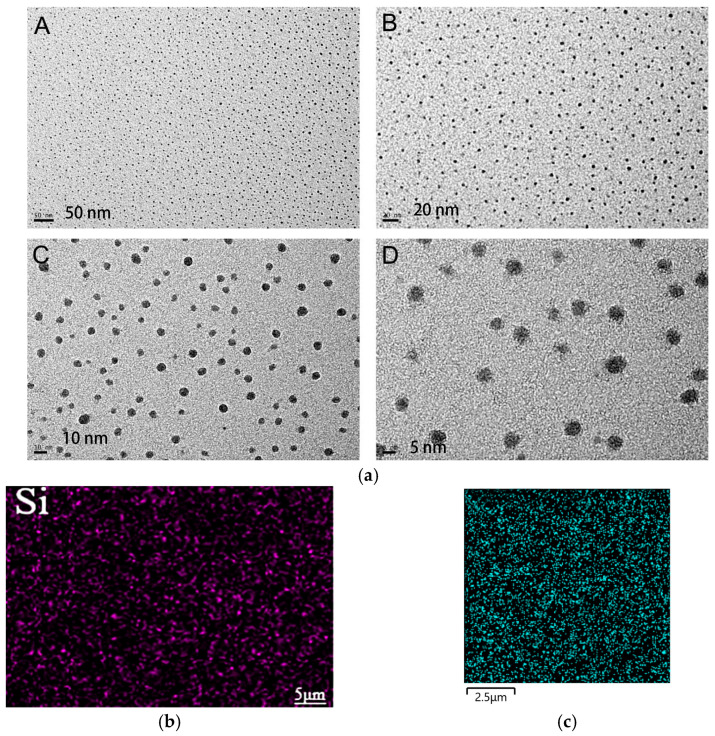
(**a**) High-resolution transmission electron microscopy (HRTEM) micrographs of composites incorporating 30 wt% OPS-BZ (scale bars for **A**: 50 nm; **B**: 20 nm; **C**: 10 nm; and **D**: 5 nm) [62]. Reproduced with permission from Kan Zhang, Macromolecules; published by American Chemical Society, 2013. (**b**) Silicon element distribution map of composites with 20 wt% EPPOSS added [64]. Reproduced with permission from Xiaodan Li, Polymer Bulletin; published by Springer Nature, 2023. (**c**) Silicon element distribution map of composites incorporating 2 wt% DDSQ-EP [64]. Reproduced with permission from Xiaoyi Sun, Polymers; published by MDPI, 2022.

Doping nanomaterials into polymers is a common and effective method to modify polymers’ optical, thermal, electrical, magnetic, and mechanical properties. In the context of improving the low dielectric properties of benzoxazines, commonly utilized nanomaterials include polyhedral oligomeric silsesquioxane (POSS), graphene (GO), and silicon dioxide (SiO_2_). POSS nanoparticles have been utilized to enhance the dielectric properties of polymers due to their distinct structure, exceptional thermal stability, monodisperse molecular weight, low dielectric constant, and adaptable molecular design [60]. Sethuraman et al. developed a benzoxazine-based nanocomposite with low dielectric properties by designing and synthesizing an allyl-capped benzoxazine and a thiol-functionalized polyhedral oligomeric sesquicarbazone (SH-POSS) (Figure 8a). The composite material was fabricated via a combination of photopolymerization and thermal curing, involving thiol–alkene reactions under ultraviolet (UV) irradiation and ring-opening polymerization of the benzoxazine ring. When 50 wt% of SH-POSS was added, the composite exhibited a dielectric constant of 2.0 at 1 MHz. The decrease in dielectric constant was attributed to the creation of voids and increased free volume facilitated by the rigid and bulky nature of SH-POSS. Additionally, the low-polarity Si−O−S bond also contributed to the reduction in dielectric constant. Li et al. [61] synthesized a benzoxazine-modified POSS(BZPOSS) (Figure 8a) and copolymerized it with epoxy resin. Incorporating 20 wt% of BZPOSS resulted in a minimum dielectric constant of 2.28 in the composites. Zhang et al. [62] prepared nanocomposites by synthesizing a benzoxazine-modified POSS (OPS-BZ) (Figure 8a) containing benzoxazole and copolymerizing it with benzoxazine. The nanocomposites exhibited a dielectric constant of 2.15 at 1 MHz with 30 wt% OPS-BZ content. The rigid benzoxazole moiety increased intermolecular distance, increased free volume, and reduced inter-nanoparticle agglomeration (Figure 9a). Li et al. [63] synthesized an epoxy-modified POSS(EPPOSS) (Figure 8a) and copolymerized it with bisphenol A cyanate (BADCy) and bisphenol A benzoxazine (BA-a) (Figure 9b). The dielectric constant decreased to 2.72 with 15 wt% EPPOSS, but severe agglomeration occurred at 20 wt%, leading to decreased cross-linking density and increased dielectric constant. Sun et al. [64] synthesized a bifunctional epoxy group modified POSS (DDSQ-EP) (Figure 8b) and doped it into benzoxazine to prepare nanocomposites (Figure 9c). Incorporating 4 wt% of DDSQ-EP reduced the dielectric constant from 3.75 to 3.45, with good dispersion of DDSQ-EP in the composite.

**Figure 10:**
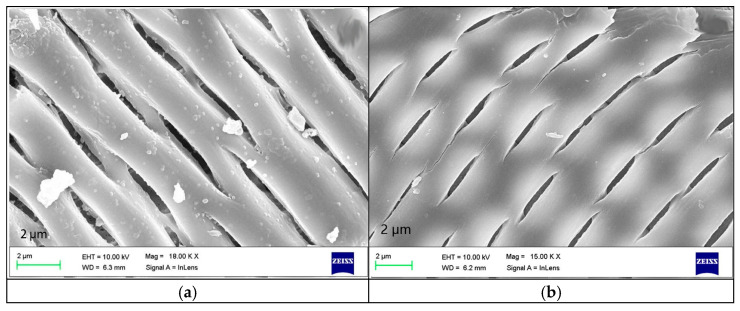
Scanning electron microscopy (SEM) images of biosilica/Chal-Bz/CrAb (**a**) and biosilica/Chal-Bz/EuAb (**b**) composites reinforced by adding 10 wt% biosilica [65]. Reproduced with permission from Arumugam Hariharan, Journal of Polymers and the Environment; published by Springer Nature, 2019.

Hariharan et al. [65] synthesized two nanocomposites by incorporating GPTMS-modified rice husk ash as biosilica into bio-based benzoxazine co-monomers: biosilica/Chal-Bz/CrAb and biosilica/Chal-Bz/EuAb. Adding 10 wt% biosilica at 1 MHz reduced the dielectric constants to 2.1 and 2.3 for the biosilica/Chal-Bz/CrAb and biosilica/Chal-Bz/EuAb composites, respectively. SEM analysis revealed a fabric-continuous weft structure in both composites, with the biosilica-reinforced composites exhibiting rough surfaces, silica nanoparticle clusters, and nanometer-sized pores (Figure 10). These findings highlight the ability of biosilica to diminish dipole–dipole interactions, lower surface energy via Si−O−Si bonds, and decrease dipole moment density via the presence of pores. As a result, the composites exhibit a low dielectric constant and reduced water absorption. Latha et al. [66] also utilized GPTMS-modified biosilica and synthesized benzoxazines (C-ima and BF-ima) using imidazolium nucleated monoamine (ima) with cashew phenol (C) and bisphenol-F (BF), respectively. Nanocomposites were prepared by incorporating the biosilica into C-ima and BF-ima and subsequent copolymerization. Adding 10 wt% biosilica resulted in a reduced dielectric constant of 2.3 at 1 MHz. Hariharan et al. [67] synthesized nanocomposites by combining cashew phenol, formaldehyde, and three different amine sources (aniline (CrAb), N,N-dimethylaminopropylamine (CrDb), and caprolactam-modified N,N-dimethylaminopropylamine (CrCb)). Three benzoxazines were synthesized, and biosilica was doped into these benzoxazines during copolymerization. Adding 10 wt% biosilica reduced the dielectric constants to 1.92 and 1.89 at 1 MHz for the CrDb-based and CrCb-based nanocomposites, respectively.

**Figure 11:**
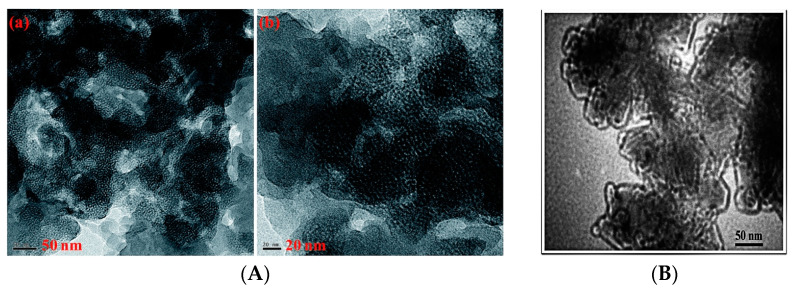
(**A**) HRTEM image of composites with 7 wt% BTMS added, scale bars for (**a**): 50 nm; (**b**): 20 nm [70]. Reproduced with permission from Kumar, Ramachandran Sasi, RSC Advances; published by Royal Society of Chemistry, 2015. (**B**) Transmission electron microscope (TEM) image of the composite with 5 wt% TSBA-15 added [72]. Reproduced with permission from Vaithilingam Selvaraj, RSC Advances; published by Royal Society of Chemistry, 2015.

MCM-41, a versatile mesoporous silica material, shows potential in improving the dielectric properties of polymers via the development of porous structured polymer composites [68]. The nano-voids present in porous silica can accommodate macromolecules, leading to enhanced polymer–nanoparticle interactions [69]. This, in turn, reduces dielectric loss. Silane coupling agents such as 3-Aminopropyltrimethoxysilane (3-APTMS) or 3-aminopropyltriethoxysilane (3-APTES) can be employed to strengthen the interactions between polymers and nanomaterials. The amino groups present in both 3-APTMS and 3-APTES enable the synthesis of benzoxazines, further enhancing the binding of benzoxazines to inorganic nanofillers. Sasi Kumar et al. [70] synthesized a benzoxazine-capped polydimethylsiloxane (PDMS-Bz) structure and benzoxazine-capped mesoporous MCM-41 silica (BTMS). They doped BTMS into PDMS-Bz and prepared composites via copolymerization. The addition of 7 wt% BTMS decreased the dielectric constant of the composites to 2.06 at 1 MHz frequency. However, further addition of BTMS resulted in an increased dielectric constant due to nanoparticle agglomeration. The incorporation of mesoporous BTMS effectively increased the porosity of the composites (Figure 11A), contributing to a reduction in the dielectric constant. Kurinchyselvan et al. [71] successfully synthesized two benzoxazines, BFCL-PBz and BSCL-PBz, along with benzoxazine-functionalized mesoporous MCM-41 silica (APMS). These benzoxazines were based on cashew nut phenol (CL) and two types of diamines: bisphenol-AF (BF)-based diamine and bisphenol-S (BS)-based diamine. The researchers copolymerised APMS with each of these two benzoxazines to prepare two nanocomposites, APMS/BFCL-PBz, and APMS/BSCL-PBz. Remarkably, the dielectric constants of these composites, APMS/BFCL-PBz and APMS/BSCL-PBz, were found to be 1.78 and 2.16, respectively, at a frequency of 1 MHz, when 7.5 wt% of APMS was added. This reduction in dielectric constant can be attributed to the long alkyl chains in cashew nut phenol, which lower the molecular polarity, and the increased porosity of the composites due to the presence of APMS. Notably, fluorine atoms in APMS/BFCL-PBz further diminish the molecular polarity, resulting in an even lower dielectric constant of 1.78. Selvaraj et al. [72] synthesized a benzoxazine (CBz) using cashew phenol and caprolactam and also prepared thiol-functionalized mesoporous silica (TSBA-15), which is similar to MCM-41. They incorporated TSBA-15 into the copolymerization process of CBz to form nanocomposites. The addition of 5 wt% TSBA-15 resulted in a decrease in the dielectric constant to approximately 2 at 1 MHz. The presence of alkyl chains from caprolactam and cashew phenol contributed to a reduction in the molecular polarity of the composites. The TSBA-15 exhibited a long chain-like structure within the composites (Figure 11B), which was attributed to the cylindrical pores of TSBA-15 and the linking effect of thiol groups. This long chain-like structure with ordered cylindrical pores created free volume, leading to a significant reduction in the dielectric constant of the composites.

Asrafali et al. [73] synthesized two benzoxazines with nitrile and carbonyl groups and prepared nanocomposites by incorporating 5 wt% nano-SiO_2_ into each benzoxazine. The addition of nano SiO_2_ reduced the composites’ dielectric constants to 2.6 and 3.2 at 1 MHz, respectively. The strong surface energy and large contact area of nano SiO_2_ facilitated a strong interaction with the polymer matrix, limiting the chain movement of polymer molecules and effectively increasing the cross-linking density, which ultimately led to a reduction in the dielectric constant of the composites.

Graphene oxide (GO) has demonstrated its potential in preparing low dielectric nanocomposites, as revealed by research findings [74,75]. Kurinchyselvan et al. [76] synthesized a benzoxazine coupling agent using cashew phenol, 3-aminopropyltriethoxysilane, and paraformaldehyde to prepare functionalized GO-C-aps. They also prepared nanocomposites (GO-BAF-a) by incorporating GO-C-aps into a copolymerization process of fluorinated benzoxazine (BAF-a) derived from bisphenol-AF, aniline, and paraformaldehyde. The addition of 10 wt% GO-C-aps resulted in reduced dielectric constants below 2.4 and a dielectric loss around 0.007. The presence of cashew phenol-based benzoxazines with long alkyl chains and graphene oxide significantly reduced polarization. Furthermore, the sp and sp^2^ carbon atoms in graphene oxide also contributed to reducing polarization behavior. Collectively, these factors contributed to the low dielectric constant observed in the composites.

The incorporation of nanofillers in benzoxazines significantly reduces the dielectric constant of polybenzoxazines. This reduction is attributed to the increased porosity of the composites caused by the nanoparticles and strong interaction forces between the nanoparticles and polymer molecules that limit the movement of polar groups. The low polarity of the nanoparticles themselves also contributes to the decrease in the dielectric constant. However, careful attention must be given to nanoparticle dispersion in the polymer as nanoparticles tend to agglomerate due to their high surface tension. This agglomeration leads to increased dielectric loss (interfacial loss) and adversely affects the mechanical properties of the composites, thus limiting their application. To address this issue, coupling agents can be introduced into benzoxazines or directly bonded to nanoparticles to enhance the bonding between nanoparticles and polybenzoxazines, as well as improve nanoparticle dispersion in polybenzoxazines.

## 7. Summary and Outlook

This paper presents a comprehensive review of low dielectric benzoxazine-based materials and examines the molecular design of fluorinated benzoxazines, bio-based benzoxazines, and other types, as well as the development of low dielectric benzoxazine-based copolymers and nanocomposites. These advancements have greatly improved the low dielectric properties of polybenzoxazines, thus they have broad application prospects in high-end electronic information materials, particularly in the manufacturing of high-integration, high-frequency, and high-speed electronic communication devices. However, challenges persist in balancing dielectric properties and other essential characteristics such as mechanical strength, heat resistance, and processability. Additionally, the understanding of the structure–property relationship in low dielectric polybenzoxazines and benzoxazine-based composites remains incomplete. Further research should explore multifunctional low dielectric benzoxazine-based composites with flame retardancy, thermal conductivity, and other desirable properties to expand their application areas. In addition, as environmental concerns escalate, the development of environmentally friendly and green low dielectric bio-based benzoxazine materials becomes increasingly important. Integrating low dielectric benzoxazine-based materials with energy storage, intelligent sensing, and biocompatibility research holds tremendous potential. In conclusion, the research and development of low dielectric benzoxazine-based materials offer broad prospects and significant potential for the future.

## Data Availability

Data availability is not applicable to this article as no new data were created or analyzed in this study.
